# Indatuximab ravtansine (BT062) combination treatment in multiple myeloma: pre-clinical studies

**DOI:** 10.1186/s13045-016-0380-0

**Published:** 2017-01-11

**Authors:** Kurt Schönfeld, Chantal Zuber, Jan Pinkas, Thomas Häder, Katrin Bernöster, Christoph Uherek

**Affiliations:** 1 Biotest AG, Landsteinerstraße 5, 63303 Dreieich, Germany; 2 ImmunoGen Inc., 830 Winter Street, Waltham, 02451-1477 MA USA

**Keywords:** Multiple myeloma, Pre-clinical, Indatuximab ravtansine, Drug combination, Tumour regression

## Abstract

Indatuximab ravtansine is a monoclonal antibody-linked cytotoxic agent that specifically targets CD138-expressing cells. Monotherapy has been shown to significantly inhibit multiple myeloma tumour growth in vivo and improve host survival. Here, we show that in most cell lines tested, indatuximab ravtansine acts additively or even synergistically with clinically approved therapies for treatment of multiple myeloma. In addition, in vivo mouse xenograft models confirmed the activity of indatuximab ravtansine in combination with lenalidamide and lenalidomide/dexamethasone. Indatuximab ravtansine may therefore be a suitable combination partner for multiple myeloma, and a clinical study is ongoing.

**Electronic supplementary material:**

The online version of this article (doi:10.1186/s13045-016-0380-0) contains supplementary material, which is available to authorized users.

## Letter

**Fig. 1:**
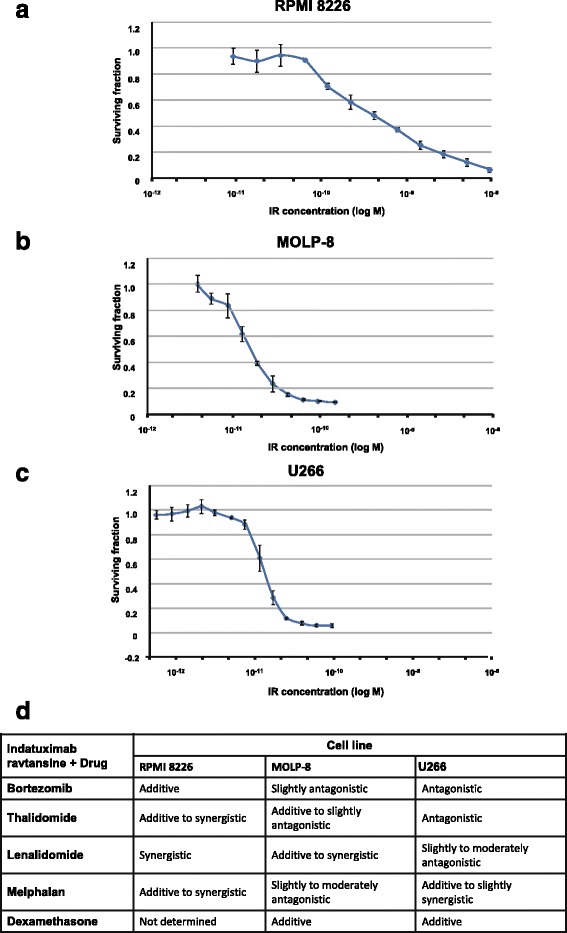
Cytotoxic effects of indatuximab ravtansine. **a** Sensitivity of RPMI 8226, **b** MOLP-8 and **c** U266 cells to indatuximab ravtansine (IR; 1 pM–100 nM) was determined by Alamar Blue proliferation assay and expressed as survival fractions. **d** Drug combinations of indatuximab ravtansine with bortezomib, thalidomide, lenalidomide, melphalan and dexamethasone

Multiple myeloma is a highly aggressive malignancy characterised by the clonal proliferation of plasma cells in the bone marrow and associated organ damage resulting from the presence of monoclonal proteins (M-proteins) in the blood or urine. The cell surface heparan sulphate proteoglycan CD138 (syndecan-1) is a transmembrane protein receptor for the extracellular matrix (ECM) that mediates cell-cell adhesion via interactions with heparan-binding molecules. In multiple myeloma, CD138 has been shown to be a co-receptor for multiple myeloma growth factors [1]. CD138 is overexpressed on malignant plasma cells and is used as a primary diagnostic marker for multiple myeloma [2]. Indatuximab ravtansine (BT062) is an antibody-drug conjugate based on a murine/human chimeric form of B-B4 (specific for CD138), linked to the maytansinoid drug DM4 by disulphide bonds and has previously been shown to significantly inhibit multiple myeloma tumour growth in vivo and to prolong host survival in xenograft mouse models of human multiple myeloma [3]. However, treatment of multiple myeloma typically involves combination therapy [4–6]. Since indatuximab ravtansine has a unique mode of action that is different to that of standard of care therapies, it might be a suitable combination partner with approved drugs for the treatment of multiple myeloma. Therefore, the effects of indatuximab ravtansine in combination with some clinically approved therapies for multiple myeloma were investigated in both in vitro and in vivo models (Additional file 1: Methods). In vitro, anti-tumour-effect studies in RPMI 8226, MOLP-8 and U266 cell lines demonstrated significant CD138 expression and sensitivity to indatuximab ravtansine (Fig. 1a–c, Additional file 2: Figure S1; IC_50_ 200 pM, RPMI 8226; 40 pM, MOLP-8; 20 pM, U266). Further in vitro studies investigated the cytotoxic effects of potential drug combinations. Additive or synergistic effects were observed for indatuximab ravtansine in combination with bortezomib, thalidomide, lenalidomide, melphalan or dexamethasone in vitro in most cell lines (Fig. 1d).

**Fig. 2:**
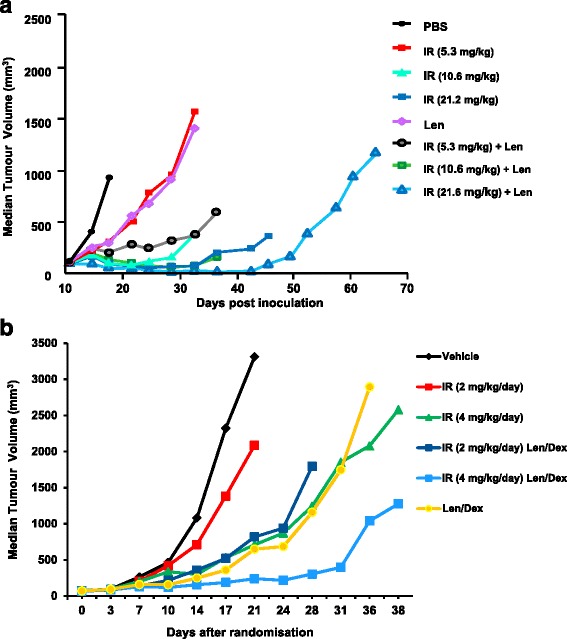
Anti-tumour activity in MOLP-8 and MMXF L363 tumours. **a** Dose-response anti-tumour activity (median tumour volume) in female CB.17 SCID mice inoculated with MOLP-8 multiple myeloma xenografts with control PBS; or indatuximab ravtansine (IR; 5.3, 10.6 or 21.2 mg/kg body weight); or lenalidomide (Len; 100 mg/kg/day); or combination of indatuximab ravtansine plus lenalidomide. Anti-tumour activity was evaluated by comparison of maximum tumour volume inhibition compared to control. **b** Anti-tumour activity (median tumour volume) in female CB.17 SCID mice inoculated with plasma cell leukaemia model MMXF L363 multiple myeloma xenografts with control (PBS); or indatuximab ravtansine (IR; 2 or 4 mg/kg/day); or lenalidomide (Len; 20 mg/kg/day) and dexamethasone (1.25 mg/kg/day); or combination of indatuximab ravtansine plus lenalidomide and dexamethasone

Mouse xenograft models (MOLP-8 and MMXF L363) were then used to investigate in vivo the anti-tumour activity of combination therapy with indatuximab ravtansine and clinically approved myeloma drugs. In MOLP-8 xenograft mouse models, indatuximab ravtansine exhibited a dose-response effect on tumour regression and this effect was enhanced when assessed in combination with lenalidomide. Lenolidamide (and later in combination with dexamethasone) was chosen for in vivo studies based on the in vitro results and due to it being an established, clinically approved treatment for multiple myeloma. The greatest effects on MOLP-8 tumour regression were observed with 21.2 mg/kg/day indatuximab ravtansine and 100 mg/kg/day lenalidomide (Fig. 2a, Additional file 3: Table S1).

The anti-tumour activity of indatuximab ravtansine was also investigated in combination with both lenalidomide and dexamethasone in an aggressive xenograft model using the plasma cell myeloma cell line MMXF L363. In this xenograft model, indatuximab ravtansine treatment alone (2 and 4 mg/kg), as well as the combination of lenalidomide and dexamethasone resulted in tumour growth delay (Fig. 2b). When assessed alone, single-agent indatuximab ravtansine at a dose of 4 mg/kg achieved similar anti-tumour activity as the combination of lenalidomide and dexamethasone. Furthermore, a stronger effect on tumour growth was observed when indatuximab ravtansine 4 mg/kg was combined with lenalidomide and dexamethasone (Fig. 2b). Treatment with indatuximab ravtansine was well tolerated.

Single-agent indatuximab ravtansine has already been shown to have clinical activity in patients with relapsed/refractory multiple myeloma [7, 8]. These pre-clinical data provide a basis for the development of indatuximab ravtansine in combination with clinically approved anti-myeloma drugs such as lenalidomide and dexamethasone and in light of these results, a clinical phase I/IIa study has been initiated to evaluate the safety and efficacy of indatuximab ravtansine in combination with lenalidomide and dexamethasone in patients with relapsed/refractory multiple myeloma. Promising initial results from this study have been reported [9], and the trial is currently ongoing.

## Additional files

Additional file 1: Methods. (DOCX 20 kb)
Additional file 2: Figure S1. CD138 expression. (PDF 134 kb)
Additional file 3: Table S1. Dose-response relationship of MOLP-8 tumours to indatuximab ravtansine alone, lenalidomide alone and combination therapy. (DOCX 12 kb)
