# Supplementary material for: Indatuximab ravtansine (BT062) combination treatment in multiple myeloma: pre-clinical studies
Source: J Hematol Oncol. 2017 Jan 11;10:13. doi: 10.1186/s13045-016-0380-0 (PMC5225632; doi:10.1186/s13045-016-0380-0)
Supplement: Additional file 1: — Methods. (DOCX 20 kb) [file 13045_2016_380_MOESM1_ESM.docx]

**Methods**

***In vitro studies of indatuximab ravtansine with conventional multiple myeloma drugs***

*Cell culture*

The MOLP-8 human multiple myeloma cell line was obtained from German Collection of Microorganisms and Cell Culture (DSMZ, Germany). RPMI 8226 and U266 human multiple myeloma cell lines were obtained from American Type Culture Collection (ATCC, Rockville, MD). Cells were cultured and tested for cytotoxicity in RPMI-1640 medium (Invitrogen, Inc, Carlsbad, CA) supplemented with 10% foetal bovine serum (FBS, RPMI 8226 cells), 15% FBS (U266 cells) or 20% FBS (MOLP-8 cells), and 50 µg/mL gentamycin (Invitrogen, Inc, Carlsbad, CA).

*Binding assay*

Cells in the exponential growth phase were used in these experiments. Cells (5x10^4^ per sample) were incubated with indatuximab ravtansine in 0.2 mL FACS buffer (2% normal goat serum in RPMI-1640 medium) on ice for 2 h, washed x1 with 0.2 mL ice-cold FACS buffer and re-suspended in 0.1 mL ice-cold FACS buffer containing 1:100 dilution of goat anti-human Fc-specific FITC-labelled antibody (Jackson Immuno Research Laboratories, West Grove, PA). After 40 min incubation on ice, cells were washed with FACS buffer, fixed in 2% formaldehyde in phosphate buffered saline (PBS) and assayed on FACS.

*Drug combination cytotoxicity assays*

Cells in the exponential growth phase were used in these experiments. Each sample was run in triplicate and each experiment repeated two or three times.

The cytotoxic activity of indatuximab ravtansine, thalidomide, lenalidomide, melphalan, and combinations of indatuximab ravtansine with these drugs were analysed as follows: 3000 cells per well (cell lines RPMI 8226 and U266) or 5000 cells per well (MOLP-8) were seeded into flat-bottom 96-well plates in 0.1 mL medium. 0.1 mL of indatuximab ravtansine (10^-12^ – 10^-7^ M) alone or single drug alone (melphalan 10^-8^ – 10^-4^ M, lenalidomide 10^-8^ – 10^-2^ M, thalidomide 5x10^-5^ – 10^-3^ M) or a combination of indatuximab ravtansine and drug were added and plates incubated for 5 days and the Alamar Blue proliferation assay was performed as described below.

A different procedure was used to measure the cytotoxic activity of bortezomib alone and bortezomib with indatuximab ravtansine, as follows: cells (quantities as above) were seeded into fibronectin-coated 96-well plates (R&D System, Minneapolis, MN) in 0.1 mL growth medium. Plates were centrifuged at 300 g for 5 min and incubated at 37^o^C under 5% CO_2_ for 24 h to immobilise the cells on the plate surface. Drugs were added (indatuximab ravtansine [10^-12^ – 10^-7^ M], bortezomib [10^-9^ – 10^-7^ M]) and plates incubated at 37^o^C under 5% CO_2_ for 24 h. After incubation, plates were centrifuged as before, drug-containing medium removed and replaced with 0.2 mL fresh growth medium. Cells were incubated for a further 5 days and the Alamar Blue proliferation assay was performed as described below.

*Alamar Blue proliferation assay*

The Alamar Blue proliferation assay was performed on all cultures in accordance with the manufacturer’s recommendations as follows: 20 µL Alamar Blue (Invitrogen, Carlsbad, CA) was added per well, plates incubated at 37^o^C under 5% CO_2_ for 18 h and fluorescence measured with excitation wavelength 530‒560 nm and emission wavelength of 590 nm.

The results were expressed as survival fractions (the ratio of the signal generated by viable cells in the treated cell population to that in the untreated cell population).

*Calculations and analyses*

*Cytotoxicity studies:* results were expressed as survival fractions (the ratio of the signal from viable cells in a treated population to that of the untreated population).

*Median effect analysis:* IC_50_ values were calculated for each drug and cell line. The IC_50_ ratios were also calculated for each drug combination. Cells were then exposed to the above mentioned dilution series of drugs either alone or in combination, with dilutions being chosen in order to generate the entire dose-effect curve. The degree of synergism, additivity or antagonism of drug combinations was determined by calculation of Combination Indexes (CI) where a CI <1 indicates synergy, CI=1 indicates additivity and CI >1 indicates antagonism [1], (CompuSyn Software Version 2004).

***Dose response anti-tumour activity of indatuximab ravtansine in combination with lenalidomide in MOLP-8 tumours in female SCID mice***

*Tumour cell line*

The MOLP-8 cell line (DSMZ, no. ACC 569) is a human multiple myeloma cell line expressing CD138. MOLP-8 cells were maintained in RPMI-1640 medium containing 2 mM L-glutamine supplemented with 10% heat-inactivated FBS and gentamicin sulfate (50 µg/mL) in a 37^o^C, 6% CO_2_-humidified chamber. Tumour cell viability upon harvest for inoculation was 95%.

*In vivo MOLP-8 human multiple myeloma xenograft model*

Female CD.17 SCID mice (Charles River Laboratories, Wilmington, MA) were inoculated *via* subcutaneous (s.c.) injection with MOLP-8 cells (1.5 × 10^7^/mouse) in a mixture of serum-free media and Matrigel (BD Biosciences, San Jose, CA). Mice were randomised by tumour volume 10 days post cell inoculation (mean tumour volume 108.9 mm^3^, SD ± 23.2) and treated with study drug by bolus intravenous (i.v.) injection on day 11 as follows.

Indatuximab ravtansine at a dose of 5.3 mg/kg, 10.6 mg/kg or 21.2 mg/kg body weight or PBS was administered as a single i.v. injection. Dose volumes were calculated according to individual animal body weights (range 0.13‒0.17 mL/mouse). Lenalidomide at 20 mg/mL in 1% CMC (at a dose of 100 mg/kg/day) was administered *via* intraperitoneal injection daily for 5 days over a 2 week period (on days 11‒15 and 18‒22 post inoculation). Dose volumes were calculated according to individual animal body weights (range 0.08‒0.11 mL/mouse/day).

Mouse body weight was monitored as a sign of toxicity, and tumours were measured once or twice weekly in three dimensions using a calliper. The tumour volume (V) was expressed in mm^3^ using the formula V = length × width × height × 0.5. Mice were monitored and sacrificed according to federal animal welfare guidelines.

*Calculations and analyses*

*Tumour regression:* Partial regression (PR) was defined as a reduction of 50% or greater in tumour volume, complete regression (CR) was defined as no palpable tumour, and tumour-free survival (TFS) as the number of mice tumour-free at the end of the study (77 days post inoculation).

*Anti-tumour activity:* This was evaluated by comparison of maximum tumour volume inhibition (study drug groups) to that in the control group.

*Relative tumour volume (RTV):* Evaluated for individual tumours according to the formula RTV_x_ [%] =T_x_ / T_0_ x 100, where T_x_ is the tumour volume on day X and T_0_ is the tumour volume on day 0.

*Tumour volume doubling (T_d_) / quadrupling time (T_q_):* Defined as the interval in time for a group median RTV to reach 200% or 400%, respectively.

*Tumour inhibition, Test / Control value (T/C %):* Calculated from the median RTV values on a given day (X) of treatment versus control groups according to the formula:

T/C_x_ [%] = (median RTV_x_ treatment group/median RTV_x_ control group) x 100

The minimum (i.e. optimum) T/C % value for a treatment group represents the maximum anti-tumour activity of that specific treatment.

*Drug activity ratings:* Group minimum T/C % values and individual relative tumour volumes (T_x_/T_0_) were used for activity rating. A T/C ≤42% is the minimum level for anti-tumour activity. A T/C <10% is considered as highly active [2].

*Tumour growth delay (TGD):* T and C are the median times (in days) required for the treatment group and control group, respectively, to reach the predetermined tumour size of 800 mm^3^. Calculations excluded tumour-free survivors.

*Log cell kill (LCK):* For subcutaneous growing tumours the LCK is calculated as (TGD value [days])/ (3.32 x T_d_) where TGD is the tumour growth delay value and T_d_ is the tumour doubling time. The LCK value can be converted to an activity rating according to the Southern Research Criteria, ranging from highly active to inactive.

*Statistical analysis:* For the evaluation of the statistical significance of tumour inhibition, the non-parametric Kruskal-Wallis test followed by Dunn’s method for pairwise comparisons was performed [3]. Individual RTVs were compared on days on which the optimal T/C value was achieved. By convention, p-values ≤ 0.05 indicate significant differences between groups. Statistical calculations were performed using GraphPad Prism bioanalytic software (version 5.01 for Windows, GraphPad Software, San Diego California USA, www.graphpad.com).

***Effect of indatuximab ravtansine in combination with lenalidomide and dexamethasone in the plasma cell leukaemia model MMXF L363 in SCID mice***

*Tumour cell line*

The MMXF L363 cell line (DSMZ, Braunschweig, Germany) is a human plasma cell leukaemia cell line expressing CD138. This tumour cell line xenografted into nude mice replicates the response of donor patients to standard anticancer drugs in over 90% of cases [4-7]. The tumour cell line was directly implanted into NOD/SCID mice to be used as donors. Tumour fragments were obtained from xenografts in passage two of these mice. After removal from donor mice, tumours were cut into fragments (4‒5 mm diameter) and placed in PBS containing 10% penicillin/streptomycin until s.c. implantation.

*In vivo MMXF L363 human plasma cell leukaemia xenograft model*

Recipient female NOD/SCID mice (Charles River, Sulzfeld, Germany) were anaesthetised by isoflurane inhalation. One small incision was made in the skin of the back and tumour fragments were transplanted with tweezers. Mice were randomised into study groups according to tumour volume (intended volume 50‒150 mm^3^). The day of randomisation and first dose of study drug was designated as Day 0. Treatment started when the inoculated tumour cells reached an average volume of approximately 80 mm^3^. Indatuximab ravtansine was administered by i.v. injection, lenalidomide given orally and dexamethasone administered *via* s.c. injection.

All treatments were given in a dose volume of 10 mL/kg. Vehicle control mice (Group 1) were treated with PBS once-weekly i.v. for 4 weeks. Group 2 mice received indatuximab ravtansine 2 mg/kg i.v. once-weekly for 4 weeks. Group 3 mice received indatuximab ravtansine at 4 mg/kg i.v. once-weekly for 4 weeks. Group 4 received indatuximab ravtansine 2 mg/kg/d i.v. in combination with lenalidomide (20 mg/kg/d orally) for 5 days per week for 4 weeks and dexamethasone (1.25 mg/kg/d s.c.) given once-weekly for 4 weeks. Group 5 received a similar treatment to group 4 but with indatuximab ravtansine at a dose of 4 mg/kg/d i.v. Group 6 received standard of care treatment alone with lenalidomide and dexamethasone at the above stated doses.

Mouse body weight was monitored as a sign of toxicity, and tumours were measured twice-weekly in two dimensions using a calliper. The tumour volume (V) was expressed in mm^3^ using the formula

V = (a × [b]^2^) × 0.5, where a = largest tumour diameter and b = perpendicular tumour diameter. Mice were monitored and sacrificed according to federal animal welfare guidelines.

*Calculations and analyses*: As described above.

**List of abbreviations**

ATCC, American Type Culture Collection; CI, combination indexes; CR, complete regression; FBS, foetal bovine serum; i.v., intravenous; LCK, log cell kill; PBS, phosphate buffered saline; PR, partial regression; RTV, relative tumour volume; s.c., subcutaneous; SCID, severe combined immunodeficiency; TFS, tumour-free survival; TGD, tumour growth delay.

**References**

1. Chou TC, Talalay P. Quantitative analysis of dose-effect relationships: the combined effects of multiple drugs or enzyme inhibitors. Adv Enzyme Regul. 1984;22:27–55.

2. Bissery MC, Guénard D, Guéritte-Voegelein F, Lavelle F. Experimental antitumor activity of taxotere (RP 56976, NSC 628503), a taxol analogue. Cancer Res. 1991;51(18):4845–4852.

3. Kruskal WH, Wallis WA. Use of ranks in one-criterion variance analysis. JASA. 1952;47(26):583–621.

4. Fiebig HH. Comparison of tumor response in nude mice and in the patients. In Winograd B, Pinedo H, eds. Human tumor xenografts in anticancer drug development. Berlin, Springer, 1988:25‒30.

5. Fiebig HH, Berger DP, Dengler WA, Wallbrecher E, Winterhalter BR. Combined in vitro/in vivo test procedure with human tumor xenografts for new drug development. Contrib Oncol. 1992;42: 321–351.

6. Fiebig HH, Dengler WA, Roth T. Human tumor xenografts: predictivity, characterization and discovery of new anticancer agents. In Fiebig HH, Burger AM, eds. Relevance of Tumor Models for Anticancer Drug Development. Contrib Oncol. 1999;54:29–50.

7. Fiebig HH, Burger AM: Human Tumor Xenografts and Explants. In Teicher BA ed, Tumor models in Cancer Research. 2001;113–137.
