# Supplementary material for: Indatuximab ravtansine (BT062) combination treatment in multiple myeloma: pre-clinical studies
Source: J Hematol Oncol. 2017 Jan 11;10:13. doi: 10.1186/s13045-016-0380-0 (PMC5225632; doi:10.1186/s13045-016-0380-0)
Supplement: Additional file 2: Figure S1. — CD138 expression. (PDF 134 kb) [file 13045_2016_380_MOESM2_ESM.pdf]

Figure S1.
CD138 expression.

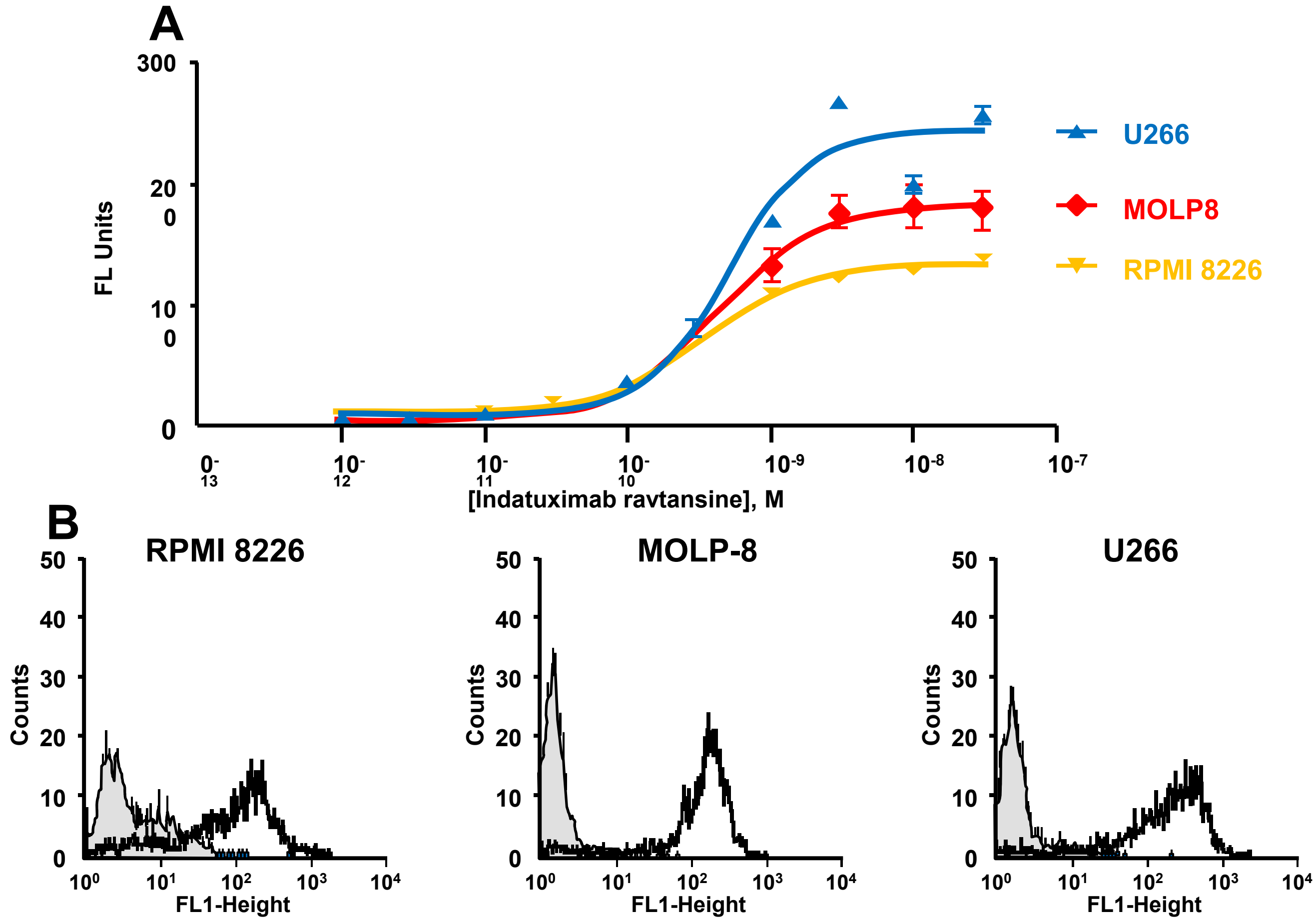

Filled: cells + secondary antibody
Empty: cells + indatuximab ravtansine ($1x10^{-8}$M) + secondary antibody

Figure S1. CD138 expression and cytotoxic effects of potential drug combinations. A. CD138 expression in U266, MOLP8 and RPMI 8226 cells was determined by FACS-based indatuximab ravtansine binding assay and data expressed as binding curves and B. FACS analysis.
