# Supplementary material for: Indatuximab ravtansine (BT062) combination treatment in multiple myeloma: pre-clinical studies
Source: J Hematol Oncol. 2017 Jan 11;10:13. doi: 10.1186/s13045-016-0380-0 (PMC5225632; doi:10.1186/s13045-016-0380-0)
Supplement: Additional file 3: Table S1. — Dose-response relationship of MOLP-8 tumours to indatuximab ravtansine alone, lenalidomide alone and combination therapy. (DOCX 12 kb) [file 13045_2016_380_MOESM3_ESM.docx]

*Table S1. Dose-response relationship of MOLP-8 tumours to indatuximab ravtansine alone, lenalidomide alone and combination therapy.*

| **MOLP-8** | **Drug treatment** | | | | | | |
| --- | --- | --- | --- | --- | --- | --- | --- |
|  | **Len alone** | **IR 5.3 mg/kg** | **IR 5.3 mg/kg + Len** | **IR 10.6 mg/kg** | **IR 10.6 mg/kg + Len** | **IR 21.2 mg/kg** | **IR 21.2 mg/kg + Len** |
| **LCK** | 0.8 | 0.7 | 1.6 | 1.5 | 2.5 | 2.5 | 3.5 |
| **T/C** | 25% | 38% | 25% | 20% | 20% | 13% | 8% |
| **PR (n)** | 0 | 0 | 0 | 0 | 2/6 | 4/6 | 5/6 |
| **CR (n)** | 0 | 0 | 0 | 0 | 0 | 1/6 | 4/6 |

Len, lenalidomide; IR, indatuximab ravtansine; LCK, log cell kill; T/C, test/control value; PR, partial remission; CR, complete remission
